# Identification of differences in gene expression implicated in the Adherent-Invasive *Escherichia coli* phenotype during *in vitro* infection of intestinal epithelial cells

**DOI:** 10.3389/fcimb.2023.1228159

**Published:** 2023-09-11

**Authors:** Queralt Bonet-Rossinyol, Carla Camprubí-Font, Mireia López-Siles, Margarita Martinez-Medina

**Affiliations:** Microbiology of Intestinal Diseases, Biology Department, Universitat de Girona, Girona, Spain

**Keywords:** Adherent-invasive *Escherichia coli*, Crohn’s disease, comparative transcriptomics, Intestine-407, arginine biosynthesis, colanic acid biosynthesis, biomarker

## Abstract

**Introduction:**

Adherent-invasive *Escherichia coli* (AIEC) is strongly associated with the pathogenesis of Crohn’s disease (CD). However, no molecular markers currently exist for AIEC identification. This study aimed to identify differentially expressed genes (DEGs) between AIEC and non-AIEC strains that may contribute to AIEC pathogenicity and to evaluate their utility as molecular markers.

**Methods:**

Comparative transcriptomics was performed on two closely related AIEC/non-AIEC strain pairs during Intestine-407 cell infection. DEGs were quantified by RT-qPCR in the same RNA extracts, as well as in 14 AIEC and 23 non-AIEC strains to validate the results across a diverse strain collection. Binary logistical regression was performed to identify DEGs whose quantification could be used as AIEC biomarkers.

**Results:**

Comparative transcriptomics revealed 67 differences in expression between the two phenotypes in the strain pairs, 50 of which (81.97%) were corroborated by RT-qPCR. When explored in the whole strain collection, 29 DEGs were differentially expressed between AIEC and non-AIEC phenotypes (p-value < 0.042), and 42 genes between the supernatant fraction of infected cell cultures and the cellular fraction containing adhered and intracellular bacteria (p-value < 0.049). Notably, six DEGs detected in the strain collection were implicated in arginine biosynthesis and five in colanic acid synthesis. Furthermore, two biomarkers based on *wzb* and *cueR* gene expression were proposed with an accuracy of ≥ 85% in our strain collection.

**Discussion:**

This is the first transcriptomic study conducted using AIEC-infected cell cultures. We have identified several genes that may be involved in AIEC pathogenicity, two of which are putative biomarkers for identification.

## 1 Introduction

Adherent-invasive *Escherichia coli* (AIEC) has been implicated in the etiology of Crohn’s disease (CD) (Baumgart et al., 2007; Eaves-Pyles et al., 2008; Martinez-Medina et al., 2009). A higher prevalence of this pathotype has been reported in CD patients compared to healthy subjects (Darfeuille-Michaud et al., 2004; Martin et al., 2004; Sasaki et al., 2007; Dogan et al., 2014); and AIEC virulence properties can explain several features of CD pathophysiology, including inflammation, mucosal translocation, and granuloma formation (Palmela et al., 2018). The AIEC pathotype is defined as *E. coli* with the ability to adhere to and invade intestinal epithelial cells (IECs) and survive and replicate inside macrophages without inducing apoptosis. AIEC strains are distinct from other *E. coli* intestinal pathotypes because the typical virulence genes of invasive pathotypes are absent in AIEC (Boudeau et al., 1999; Glasser et al., 2001; Kittana et al., 2019). Conversely, AIEC strains are phylogenetically diverse and genetically similar to extra-intestinal pathogenic *E. coli* (ExPEC) (Martinez-Medina et al., 2009; Miquel et al., 2010; Nash et al., 2010).

Currently, the genetic elements and mechanisms underlying the AIEC phenotype are not fully understood, and no molecular markers exist for its identification. Detection still relies on phenotypic assays on infected cell cultures, making it time-consuming and poorly standardized. Previous studies using molecular methods to identify genes associated with AIEC and widely distributed within the pathotype were unsuccessful (Dogan et al., 2014; Vazeille et al., 2016; Céspedes et al., 2017). Comparative genomics studies failed to identify any definitive molecular signatures for AIEC, although several putative biomarkers were proposed, with accuracies ranging from 56% to 85% (Deshpande et al., 2015; Zhang et al., 2015; Desilets et al., 2016; O’Brien et al., 2017; Camprubí-Font et al., 2018). Further analyses are required to determine the utility of these biomarkers across diverse *E. coli* strains from different geographical regions and phylogenetic backgrounds.

Previously, we proposed using three single nucleotide polymorphisms (SNPs) that, when combined, achieved an accuracy of 81% for AIEC detection (Camprubí-Font et al., 2018). However, further investigation revealed that their usefulness was limited to AIEC clones circulating in Spain and not for geographically distant strains (Camprubí-Font et al., 2020). Therefore, there remains a need to identify validated biomarkers for the molecular detection of AIEC.

In a previous study, we identified AIEC/non-AIEC strain pairs with an identical pulsotype but differing ability to invade IECs (Martinez-Medina et al., 2009). We hypothesized that differential gene expression could be responsible for the AIEC phenotype. To date, three comparative transcriptomic studies have been conducted using AIEC strains. Zhang et al. found six genes over-expressed in the AIEC LF82 strain compared to the non-pathogenic strain HS while growing in Luria-Bertani (LB), and these genes were related to bacteriophage infection and inorganic ion transport and metabolism (Zhang et al., 2015). Delmas et al. compared AIEC LF82 and non-pathogenic *E. coli* strain K-12 gene expression in the presence and absence of bile salts. The results showed that bile salts induced over-expression of six genes involved in ethanolamine utilization in LF82 but not K-12 (Delmas et al., 2019). Recently, Elhenawy et al. used the AIEC NRG875c strain as a model to evaluate differences in gene expression between *in vivo* and *in vitro* conditions. They identified the type IV secretion system as a fundamental virulence factor for *in vivo* survival (Elhenawy et al., 2021).

Although these studies have provided valuable information about AIEC pathophysiology, further research is needed to fully understand the molecular mechanisms underlying AIEC pathogenicity and to identify AIEC-specific traits widely distributed among this phenotype. Moreover, none of these studies assessed the suitability of gene expression differences as biomarkers for the molecular identification of the pathotype. In this study, we sequenced the genes expressed during IEC infection for the first time, the model used to differentiate between invasive and non-invasive *E. coli* strains *in vitro*, to identify the genes expressed during adhesion, invasion, and intracellular survival. We also used two genetically close AIEC/non-AIEC strain pairs as models for comparison, which narrowed the differences that could be found between strains and increased the chance of identifying genetic features associated with the AIEC phenotype. Finally, differentially expressed genes were further investigated in a genetically diverse strain collection of AIEC and non-AIEC strains to identify mechanisms widely used in a phylogenetically diverse AIEC collection that could serve as biomarkers.

## 2 Results

### 2.1 Comparative transcriptomics between AIEC/non-AIEC strain pairs

RNA-seq was conducted on bacterial mRNA purified extracts from the supernatant fractions of infected IECs (SN fraction) and the eukaryotic cell fractions containing adhered and invading bacteria (A/I fraction) of two AIEC/non-AIEC strain pairs. The AIEC07/ECG04 strain pair was obtained from the ileum of a control subject and the AIEC17/ECG28 strain pair was isolated from the colon of a CD patient in a previous study (Martinez-Medina et al., 2009). Sequences were trimmed, and between 16.0 and 41.9 million reads were obtained for SN fraction samples, while A/I samples ranged from 315.6 to 379.6 million reads. The percentage of reads that mapped to the AIEC UM146 reference genome ranged from 23.2 to 80.2% in SN samples and from 1.5 to 8.0% in A/I samples (**Supplementary Table 1**). Nonetheless, the coverage of the bacterial genome was similar in the SN (73.1 ± 14.8%) and A/I (85.4 ± 3.7%) fractions.

**Figure 1:**
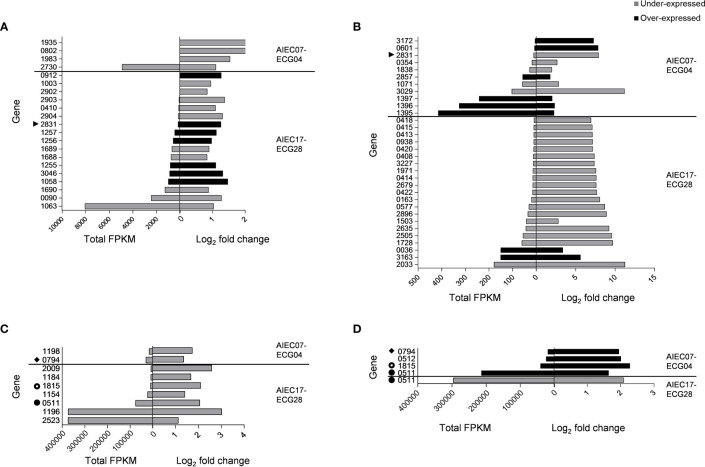
Differentially expressed genes (DEGs) displayed in comparative transcriptomics between AIEC and non-AIEC strain pairs during I-407 infection (p-value< 0.050). Total fragments per kilobase of transcript per million mapped reads (FPKM) and log2 fold change are shown. Symbols point out genes found in two or more comparisons. Genes that presented an over-expression in AIEC are shown in black, while genes that showed an under-expression are shown in grey. **(A)** DEGs found in the supernatant (SN) fraction of the strain pairs corresponding to mRNA. **(B)** DEGs found in the fraction containing infected cells with adherent and/or intracellular bacteria (A/I) of the strain pairs, corresponding to mRNA. **(C)** DEGs found in the SN fraction of the strain pairs correspond to sRNA and/or tRNA. **(D)** DEGs found in the A/I fraction of the strain pairs that correspond to sRNA and/or tRNA.

Comparative transcriptomics between the two AIEC strains and their non-AIEC counterparts revealed a total of 67 differences in gene expression, of which 48 were under-expressed in AIEC (25 in the A/I fraction and 23 in the SN fraction) and 19 were over-expressed (12 in the A/I fraction and 7 in the SN fraction) (**Figure 1**). These 67 differences in gene expression corresponded to 62 genes (i.e., differentially expressed genes, DEGs) because some DEGs were differentially expressed in more than one comparison.

Regarding the gene expression levels, total fragments per kilobase of transcript per million mapped reads (FPKM) values were lower than 1000 in 73.13% of the studied DEGs. A group of 13 genes had higher total FPKM values, of which 12 were classified as tRNA in the functional categories, and one was classified as sRNA (**Figure 1**; **Supplementary Table 2**).

### 2.2 Functional categories of the differentially expressed genes

**Figure 2:**
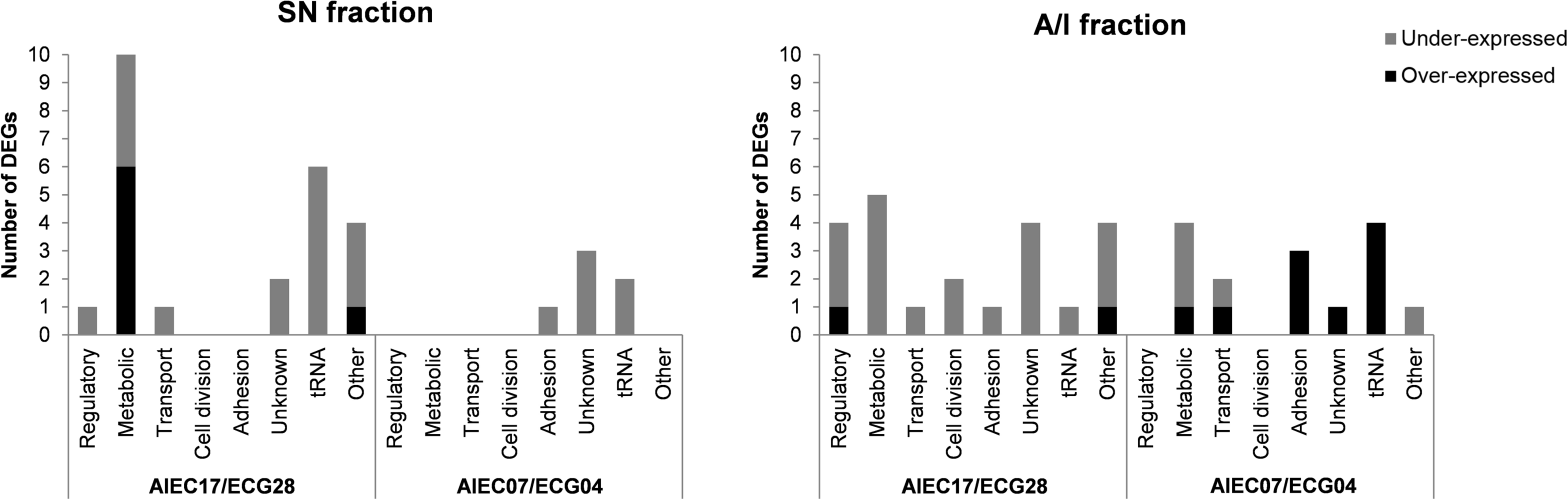
Predicted function of the differentially expressed genes (DEGs) in each comparison distributed in eight functional categories. The number of DEGs in each category is shown for the SN and A/I fractions of the two strain pairs. Genes that presented an over-expression in AIEC are shown in black, while genes that showed an under-expression are shown in grey. The DEGs of this figure are listed in **Supplementary Table 2**.

Functional analysis was conducted to classify the DEGs into eight functional categories (**Figure 2**). The detailed list of the functions of each gene is specified in **Supplementary Table 2**. Overall, the most abundant category included genes related to metabolic processes (19 genes), followed by tRNAs (13 genes) and genes of unknown function (10 genes). Genes related to adhesion (5 genes), regulatory functions (5 genes), transport (4 genes), and cell division (2 genes) were also identified. The remaining nine genes were included in the “others” category, which comprised genes related to antibiotic resistance, protein degradation, stress response, iron processing, integrases, sRNA, and rRNA.

### 2.3 Confirmation of comparative transcriptomics results using RT-qPCR

The same samples used for RNA-seq were utilized to validate the comparative transcriptomics results by means of RT-qPCR. A total of 61 differences in gene expression were analyzed because data was not obtained for six genes due to incorrect quantification, with values out of range of Fluidigm.

**Figure 3:**
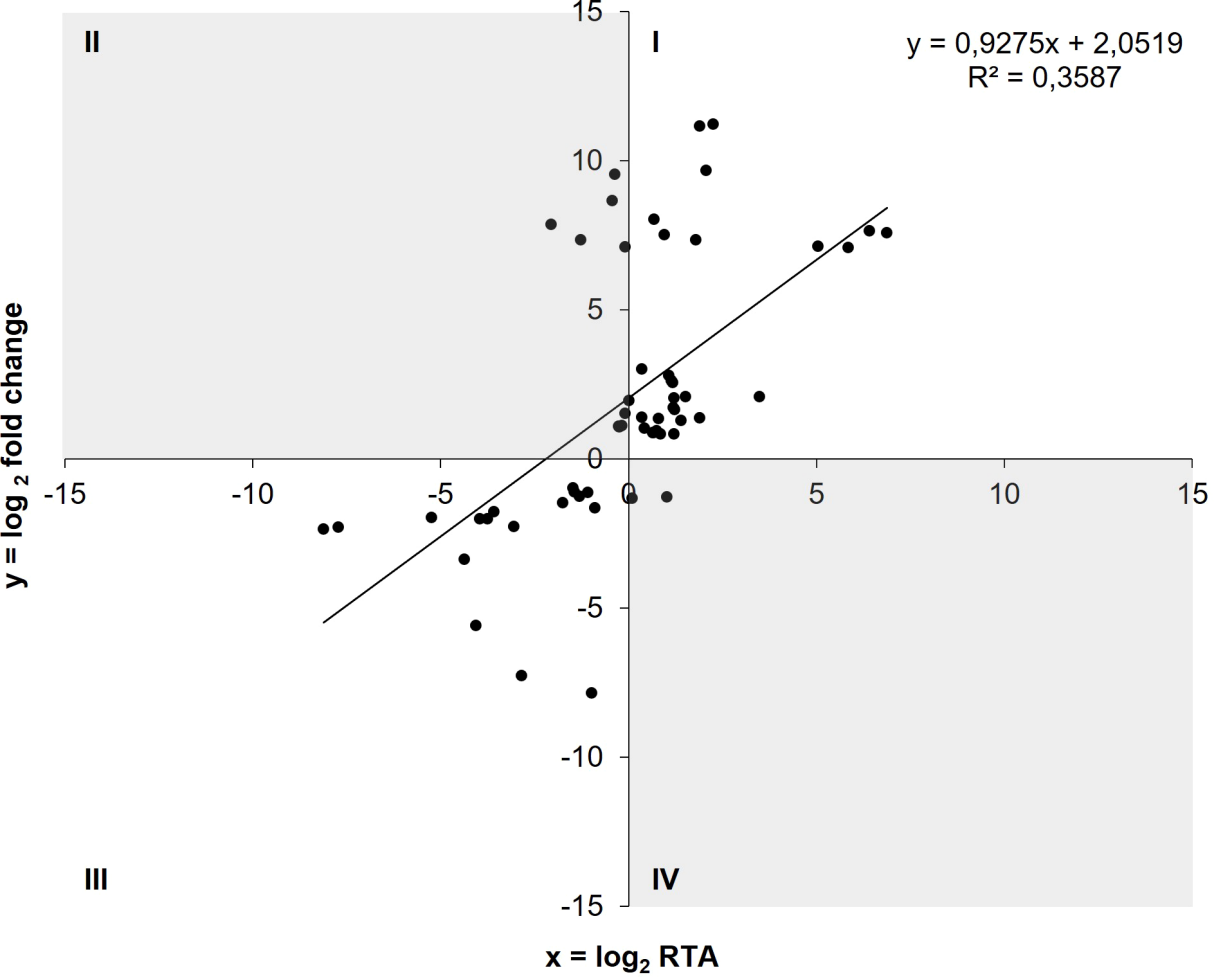
Correlation between log2 fold change values obtained in comparative transcriptomics and log2 RTA values obtained by RT-qPCR (ρ = 0.677, p<0.001). Amplification of the DEGs with RT-qPCR was done in the same samples that were sequenced. Genes located in quadrant I revealed an under-expression in AIEC in both techniques, whereas the genes in quadrant III revealed an over-expression (n = 50). Quadrants II and IV (in grey) contain genes with discrepancies between the two techniques (n = 11).

Thirty-three out of the 42 genetic AIEC under-expressions (78.57%) and 17 of the 19 genetic over-expressions (89.47%) detected in RNA-seq were confirmed by RT-qPCR as under- and over-expressions, respectively (**Figure 3**). Moreover, a positive correlation was observed between log_2_ fold change values obtained from comparative transcriptomics and log_2_ RTA (relative transcript abundance) values obtained from RT-qPCR (ρ = 0.677, p-value<0.001) (**Figure 3**). Therefore, these results confirm the reliability of RNA-seq data, and subsequent quantifications of gene expression were performed by RT-qPCR.

Non-purified samples of the strain pairs were also analyzed by RT-qPCR. These samples were obtained before the purification steps of the MICROBEnrich kit and the Ribo-Zero Magnetic Gold kit (epidemiology). The differences in gene expression between purified and non-purified samples were investigated. Gene expression differences were quantified by RT-qPCR in purified and non-purified RNA extracts from the AIEC/non-AIEC strain pairs. The log_2_ RTA values obtained from the two types of samples were compared to determine the effects of purification.

Differences in log_2_ RTA values were found between purified and non-purified samples of AIEC SN, non-AIEC SN, and AIEC A/I (p-value ≤ 0.043), whereas no statistically significant differences were found in non-AIEC A/I samples (**Table 1**). Overall, purified samples showed higher Ct values in the RT-qPCR, indicating a lower number of copies of the transcript. Nonetheless, 34 out of the 59 differences of gene expression studied (57.63%) showed a concordance of over or under-expression between purified and non-purified samples (**Supplementary Table 3**).

**Table 1:** Effect of RNA purification on gene expression quantification of the strain pairs.

Samples	Purification	Mean log_2_ RTA ± SD	p-value of paired t-test
AIEC SN	Yes	-1.099 ± 3.154	0.0003
AIEC SN	No	0.494 ± 3.438	
Non-AIEC SN	Yes	-0.760 ± 3.015	0.0432
Non-AIEC SN	No	-0.483 ± 2.918	
AIEC A/I	Yes	0.554 ± 3.504	0.0288
AIEC A/I	No	1.019 ± 2.336	
Non-AIEC A/I	Yes	0.799 ± 3.724	0.1955
Non-AIEC A/I	No	1.163 ± 2.720	

The mean log2 RTA values of 59/67 gene expression differences of purified and non-purified samples are shown for each phenotype (AIEC and non-AIEC), and a fraction (SN and A/I) since 8/67 of these differences did not show amplification values in the RT-qPCR. A paired t-test was applied in each comparison, and a p-value was given.

### 2.4 Expression levels of confirmed DEGs in the strain collection

The study of 62 DEGs, identified using RNA-seq and comparative transcriptomics, was extended to a larger collection of 14 AIEC and 23 non-AIEC strains to evaluate whether the differences in expression were widespread among AIEC strains and specific to this group. All the information related to the strains background, adhesion-invasion index, and intramacrophage survival capacities can be found in **Supplementary Table 6**. Additionally, 5 genes of interest based on bibliographic research (*fimH*, *chiA*, *adiA*, *adiC*, and *potE*) (Low et al., 2013; Charlier and Bervoets, 2019; Chervy et al., 2020), and 10 genes encoded in genomic fragments where other DEGs were identified were included in the analysis. However, five of the 62 DEGs (0090, 0802, 1503, 2635, and 2679) were excluded from the analysis in the strain collection due to incorrect amplification related to high or low concentrations of the gene. Overall, 72 gene quantifications obtained by RT-qPCR were studied in the strain collection.

**Figure 4:**
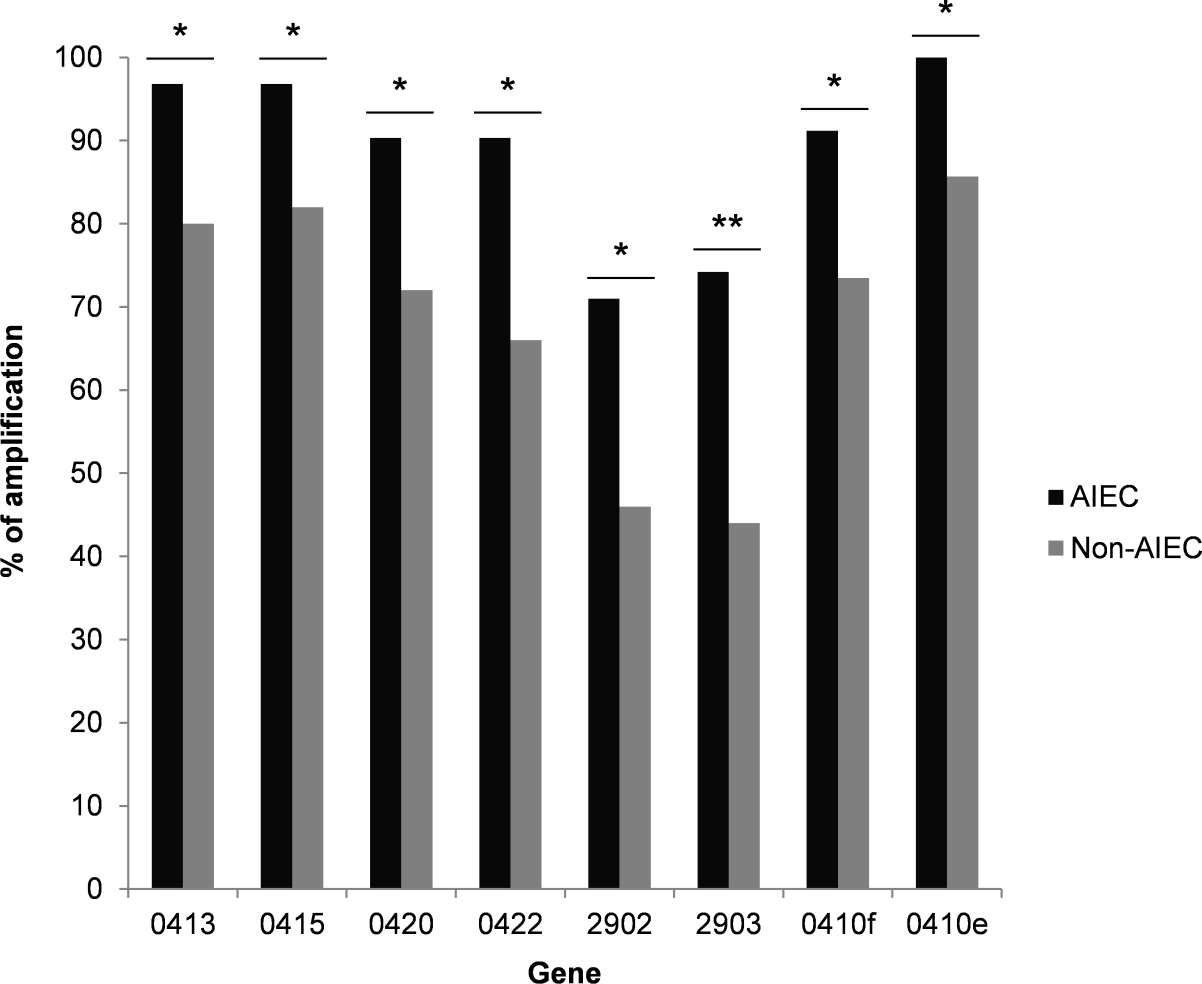
Percentage of strains amplified in a collection of clinical isolates for each DEG. Only those DEGs with statistically significant differences between AIEC and non-AIEC strains are shown. *p-value<0.05; **p-value<0.01. Genes that presented an over-expression in AIEC are shown in black, while genes that showed an under-expression are shown in grey.

Since differences in gene expression can only be compared for samples in which the genes have been amplified, a preliminary analysis was performed to study the frequency of gene amplification in AIEC and non-AIEC samples. Non-amplification can result from the absence of a gene in a given strain or an expression level below the detection limit. Eight genes (0413, 0415, 0420, 0422, 2902 and 2903, 0410f, and 0410e) were amplified more frequently in AIEC strains than in non-AIEC strains (p-value ≤ 0.047) (**Figure 4**). Despite the high percentage of amplification of these genes in AIEC strains (71-100%), they were not AIEC-specific, as these genes were also detected in a high percentage of non-AIEC strains (44-85.7%). Four genes are involved in the colanic acid biosynthesis pathway, one is a probable serine/threonine kinase, and the remaining three genes have unknown functions.

**Figure 5:**
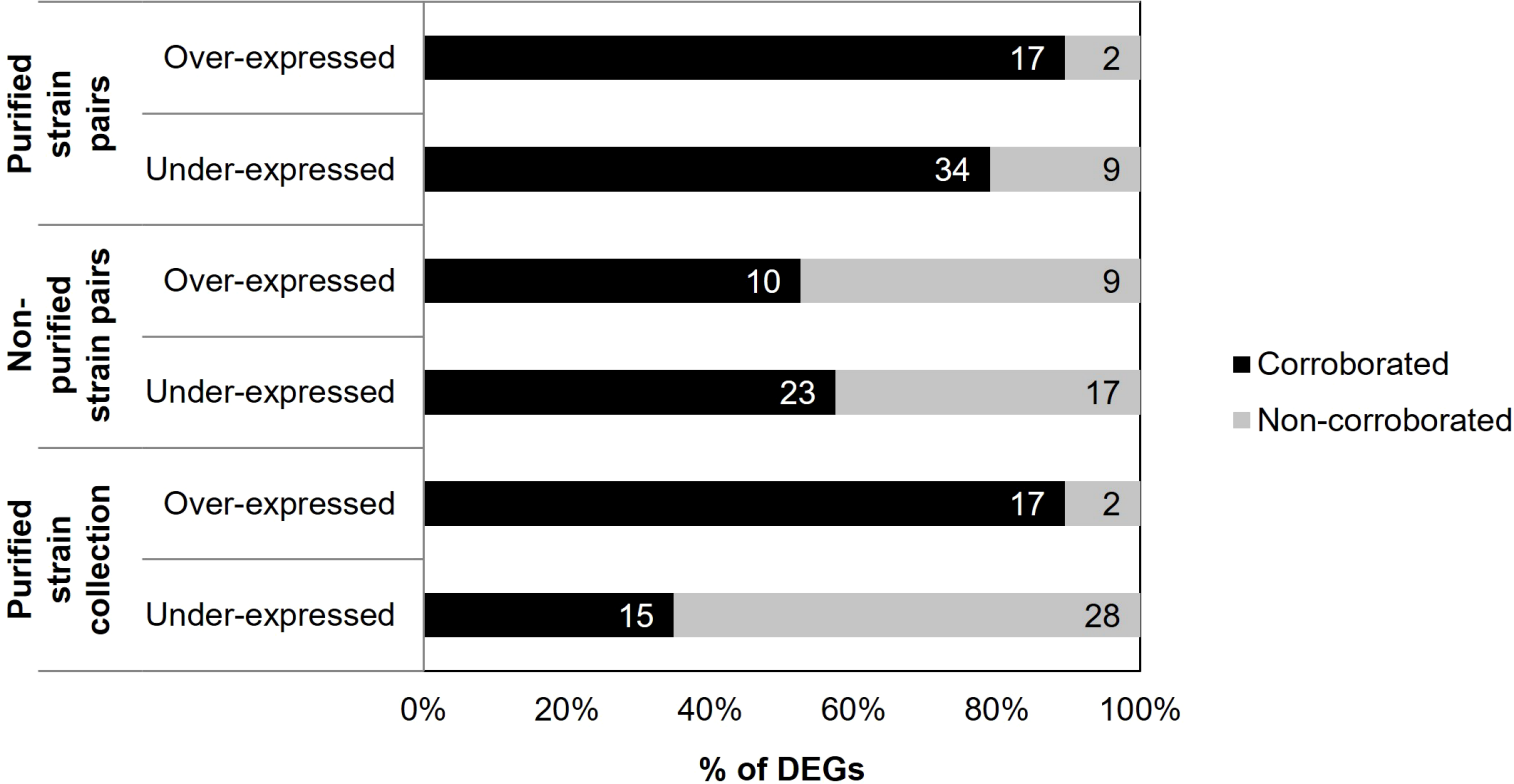
Percentage of genes corroborated for the different groups of samples performed with RT-qPCR compared with the initial results obtained by RNA-Seq. The number of DEGs is indicated on each occasion.

A concordance was found between the differential gene expression data obtained by RNA-seq and RT-qPCR data obtained from the entire strain collection (**Supplementary Table 3**). In this case, 62 out of 67 differences in gene expression were analyzed, and five were excluded because data were unavailable. Specifically, 17 of the 19 (89.47%) over-expressions and 15 of the 43 (34.88%) under-expressions found in RNA-seq were also over-expressed and under-expressed, respectively, in the non-purified samples of the strain collection studied by RT-qPCR. Thus, an overall validation of 51.61% of the differential expressions was achieved (**Figure 5**).

Statistically significant differences (p-value ≤ 0.042) between AIEC and non-AIEC strains were found for 29 genes (40.27%). Most of them (N = 26) were detected in the SN fraction, one in A/I (1184), and two (0511 and 1198) were found in both fractions (**Table 2**; **Supplementary Figure 1**). Gene expression was also compared between SN and A/I fractions within each strain group. Statistically significant differences (p-value ≤ 0.049) were found in 42 genes (58.33%); two of them were detected in AIEC, 31 in non-AIEC, and nine in both phenotypes (**Table 3**; **Supplementary Figure 1**). Most were over-expressed in the A/I fraction (N = 37), while only five genes were over-expressed in the SN fraction. The nine DEGs detected in both phenotypes were over-expressed in the same fraction in AIEC and non-AIEC (1198, 0036, 2857, 1184, 1063, 1196, 0512, 2009, and 0414d) (**Supplementary Table 4**).

**Table 2:** Values of log_2_ RTA of the genes with a statistically significant expression between AIEC and non-AIEC phenotypes are displayed for a given fraction.

ID	Gene	$\bar{x}$ log_2_ RTA AIEC A/I ± SD	$\bar{x}$ log_2_ RTA non-AIEC A/I ± SD	p-value A/I	$\bar{x}$ log_2_ RTA AIEC SN ± SD	$\bar{x}$ log_2_ RTA non-AIEC SN ± SD	p-value SN
*fimH*	*fimH*	-0.027 ± 0.552	-1.483 ± 0.511	0.0740	0.491 ± 0.941	-2.127 ± 0.595	**0.0190**
36	*tdcA*	2.925 ± 0.348	3.248 ± 0.551	0.6770	8.786 ± 0.531	5.856 ± 0.501	**0.0010**
410	*mdtB*	2.123 ± 0.236	1.588 ± 0.408	0.3570	3.841 ± 0.917	1.073 ± 0.597	**0.0200**
0410f	*pphC*	-2.221 ± 0.485	-2.703 ± 0.677	0.2450	0.620 ± 1.444	-3.695 ± 1.231	**0.0100**
413	*wza*	1.089 ± 0.214	1.317 ± 0.580	0.6490	4.000 ± 1.547	0.762 ± 1.071	**0.0350**
414	*wzb*	1.313 ± 0.250	2.167 ± 0.597	0.5580	4.622 ± 1.628	0.476 ± 0.766	**0.0080**
511	*UM146_RS07855*	2.769 ± 0.385	1.695 ± 0.255	**0.0180**	1.267 ± 0.424	-0.211 ± 0.405	**0.0310**
794	*UM146_RS12625*	4.224 ± 0.394	3.291 ± 0.356	0.1010	4.051 ± 0.843	1.987 ± 0.606	**0.0190**
912	*citC*	1.445 ± 0.154	0.633 ± 0.399	0.6740	3.877 ± 1.391	0.523 ± 0.813	**0.0200**
1058	*zraP*	0.538 ± 0.190	0.532 ± 0.374	0.9910	3.044 ± 0.896	0.408 ± 0.594	**0.0180**
1071	*nirB*	2.398 ± 0.275	1.786 ± 0.387	0.0730	3.026 ± 1.072	0.179 ± 0.407	**0.0320**
1154	*UM146_RS19150*	1.235 ± 0.423	0.459 ± 0.299	0.1370	-0.204 ± 0.949	-2.383 ± 0.516	**0.0400**
1184	*UM146_RS19670*	4.483 ± 0.397	3.205 ± 0.385	**0.0390**	0.998 ± 0.552	-0.300 ± 0.507	**0.1580**
1198	*UM146_RS19850*	5.313 ± 0.522	3.901 ± 0.316	**0.0190**	2.602 ± 0.803	0.544 ± 0.490	**0.0310**
1255	*argC*	4.154 ± 0.471	3.970 ± 0.427	10.000	3.372 ± 0.748	-0.015 ± 0.770	**0.0090**
1256	*argB*	4.317 ± 0.489	3.915 ± 0.437	0.5590	4.088 ± 0.846	0.482 ± 0.612	**0.0020**
1257	*argH*	4.501 ± 0.355	4.109 ± 0.402	0.5130	3.990 ± 0.767	1.066 ± 0.619	**0.0090**
1815	*UM146_RS05255*	2.088 ± 0.288	1.246 ± 0.374	0.1260	1.270 ± 0.716	-1.938 ± 0.524	**0.0010**
1983	*ydjM*	0.992 ± 0.179	0.923 ± 0.272	0.8340	1.627 ± 0.720	-0.781 ± 0.312	**0.0020**
2505	*cueR*	1.357 ± 0.325	0.873 ± 0.348	0.3630	2.947 ± 0.494	0.753 ± 0.445	**0.0110**
2523	*ffs*	2.158 ± 0.494	1.033 ± 0.212	0.0520	0.137 ± 0.517	-1.348 ± 0.290	**0.0140**
2831	*ilvN*	2.857 ± 0.206	2.999 ± 0.375	0.7850	4.751 ± 0.862	1.993 ± 0.511	**0.0070**
2857	*pstA*	0.887 ± 0.353	-0.087 ± 0.515	0.2030	-1.877 ± 0.594	-4.814 ± 0.464	**0.0010**
3029	*UM146_RS22055*	-0.186 ± 1.295	-0.895 ± 0.982	0.4940	1.067 ± 1.752	-5.838 ± 1.892	**0.0260**
3046	*argF*	0.757 ± 0.172	0.385 ± 0.286	0.2750	1.361 ± 0.461	0.055 ± 0.339	**0.0350**
3046b	*rraB*	0.763 ± 0.529	-0.095 ± 0.637	0.3540	-0.379 ± 1.287	-3.458 ± 0.807	**0.0420**
3046c	*yjgM*	-1.921 ± 0.311	-2.025 ± 0.413	0.4280	-1.685 ± 0.756	-3.879 ± 0.590	**0.0320**
3163	*btsT*	1.727 ± 0.260	2.338 ± 0.338	0.3190	3.954 ± 0.810	1.726 ± 0.508	**0.0240**
3172b	*yjjP*	0.249 ± 0.250	-0.202 ± 0.579	0.4800	1.614 ± 0.747	-1.087 ± 0.736	**0.0230**

P-values ≤ 0.05 were considered statistically significant and are highlighted in bold.

**Table 3:** Values of log_2_ RTA of the genes displaying statistically significant expression between SN and A/I fractions for a given phenotype.

ID	Gene	$\bar{x}$ log_2_ RTA AIEC A/I±SD	$\bar{x}$ log_2_ RTA AIEC SN ± SD	p-value AIEC	$\bar{x}$ log_2_ RTA non-AIEC A/I ± SD	$\bar{x}$ log_2_ RTA non-AIEC SN ± SD	p-value non-AIEC
*adiA*	*adiA*	-2.691 ± 0.458	0.242 ± 1.127	0.0583	-2.459 ± 0.439	-0.246 ± 0.610	**0.0255**
*chiA*	*chiA*	2.832 ± 0.384	3.481 ± 1.229	0.6521	2.305 ± 0.441	0.799 ± 0.526	**0.0171**
36	*tdcA*	2.925 ± 0.348	8.786 ± 0.531	**<0.0001**	3.248 ± 0.551	5.856 ± 0.501	**0.0007**
163	*recX*	1.104 ± 0.364	-1.645 ± 1.558	0.1418	1.188 ± 0.196	-2.312 ± 0.332	**<0.0001**
0410b	*mdtA*	-5.225 ± 0.387	0.014 ± 3.185	0.1689	-5.511 ± 0.500	-3.819 ± 2.116	**0.0285**
0410c	*yegL*	-0.489 ± 0.512	-0.63 ± 0.900	0.9616	-0.665 ± 0.682	-3.014 ± 0.944	**0.0106**
0410d	*yegI*	-0.166 ± 0.333	-2.877 ± 0.919	**0.0228**	-1.188 ± 0.350	-4.706 ± 0.738	**0.0002**
414	*wzb*	1.313 ± 0.250	4.622 ± 1.628	**0.0489**	2.167 ± 0.597	0.476 ± 0.766	**0.1413**
415	*wzc*	1.398 ± 0.166	2.878 ± 1.517	0.3412	1.592 ± 0.524	-0.702 ± 0.300	**0.0131**
418	*wcaE*	1.195 ± 0.229	3.214 ± 2.099	0.5153	1.613 ± 0.641	0.427 ± 0.863	**0.0073**
420	*gmd*	1.309 ± 0.271	2.490 ± 2.082	0.4748	1.662 ± 0.633	-0.62 ± 0.290	**0.0075**
511	*UM146_RS07855*	2.769 ± 0.385	1.267 ± 0.424	0.0892	1.695 ± 0.255	-0.211 ± 0.405	**0.0001**
512	*UM146_RS07860*	2.111 ± 0.197	0.743 ± 0.292	0.0072	1.432 ± 0.281	0.099 ± 0.251	**<0.0001**
601	*sufE*	-1.372 ± 0.126	-1.851 ± 0.435	0.5267	-1.543 ± 0.298	-2.523 ± 0.510	**0.0490**
938	*UM146_RS28105*	1.390 ± 0.211	0.715 ± 0.938	0.6247	0.831 ± 0.639	-0.806 ± 0.599	**0.0069**
1063	*UM146_RS26980*	3.809 ± 0.333	-0.369 ± 0.264	**0.0001**	3.628 ± 0.266	-0.007 ± 0.297	**<0.0001**
1071	*nirB*	2.398 ± 0.275	3.026 ± 1.072	0.6168	1.786 ± 0.387	0.179 ± 0.407	**0.0271**
1154	*UM146_RS19150*	1.235 ± 0.423	-0.204 ± 0.949	0.1328	0.459 ± 0.299	-2.383 ± 0.516	**<0.0001**
1184	*UM146_RS19670*	4.482 ± 0.397	0.998 ± 0.552	**0.0088**	3.205 ± 0.385	-0.299 ± 0.507	**0.0001**
1196	*UM146_RS19840*	4.620 ± 0.462	1.967 ± 0.464	**0.0039**	4.019 ± 0.330	1.648 ± 0.237	**<0.0001**
1198	*UM146_RS19850*	5.313 ± 0.522	2.602 ± 0.803	**0.0408**	3.901 ± 0.316	0.544 ± 0.490	**<0.0001**
1255	*argC*	4.154 ± 0.471	3.372 ± 0.748	0.5245	3.970 ± 0.427	-0.015 ± 0.770	**0.0001**
1256	*argB*	4.317 ± 0.489	4.088 ± 0.846	0.8991	3.915 ± 0.437	0.482 ± 0.612	**<0.0001**
1257	*argH*	4.501 ± 0.355	3.990 ± 0.767	0.516	4.109 ± 0.402	1.066 ± 0.619	**0.0003**
1395	*fimC*	-3.214 ± 0.849	-2.687 ± 1.457	0.9241	-3.908 ± 0.642	-5.376 ± 0.660	**0.0005**
1396	*fimD*	-1.292 ± 0.887	-3.665 ± 1.358	0.3696	-3.261 ± 0.698	-6.438 ± 0.940	**0.0005**
1397	*fimF*	-0.822 ± 0.639	-1.672 ± 1.461	0.6204	-2.150 ± 0.553	-4.285 ± 0.672	**0.0100**
1688	*srlD*	-1.204 ± 0.697	-1.408 ± 1.514	0.9467	-0.666 ± 0.539	-1.633 ± 0.695	**0.0286**
1689	*srlB*	-1.406 ± 0.765	-1.089 ± 1.387	0.7307	-1.131 ± 0.569	-2.225 ± 0.635	**0.0014**
1690	*srlA*	-1.633 ± 0.770	-0.107 ± 1.268	0.3813	-1.114 ± 0.610	-2.015 ± 0.547	**0.0192**
1815	*UM146_RS05255*	2.088 ± 0.288	1.270 ± 0.716	0.1783	1.246 ± 0.374	-1.938 ± 0.524	**<0.0001**
1838	*glpC*	1.975 ± 0.501	4.254 ± 0.699	**0.01**	2.598 ± 0.543	2.432 ± 0.539	**0.9771**
1983	*ydjM*	0.992 ± 0.179	1.627 ± 0.720	0.5102	0.923 ± 0.272	-0.781 ± 0.312	**0.0002**
2009	*UM146_RS09110*	1.382 ± 0.364	-3.452 ± 1.459	**0.0198**	0.438 ± 0.344	-5.444 ± 0.488	**<0.0001**
2523	*ffs*	2.158 ± 0.494	0.137 ± 0.517	0.0521	1.033 ± 0.212	-1.348 ± 0.290	**<0.0001**
2730	*UM146_RS28160*	1.698 ± 0.365	-0.425 ± 1.208	0.0966	1.271 ± 0.434	-2.630 ± 0.664	**<0.0001**
2857	*pstA*	0.887 ± 0.353	-1.877 ± 0.594	**0.0066**	-0.087 ± 0.515	-4.814 ± 0.464	**<0.0001**
3029	*UM146_RS22055*	-0.186 ± 1.295	1.067 ± 1.752	0.461	-0.895 ± 0.982	-5.838 ± 1.892	**0.0003**
3029c	*UM146_RS22065*	-2.804 ± 0.359	-1.406 ± 1.424	0.3673	-2.224 ± 0.755	-4.456 ± 1.898	**0.0093**
3046b	*rraB*	0.763 ± 0.529	-0.379 ± 1.287	0.6717	-0.095 ± 0.637	-3.458 ± 0.807	**0.0014**
3046c	*yjgM*	-1.921 ± 0.311	-1.685 ± 0.756	0.6531	-2.025 ± 0.413	-3.879 ± 0.590	**0.0168**
3227	*UM146_RS24370*	-0.01 ± 0.773	0.332 ± 1.973	0.6075	0.161 ± 0.858	-3.702 ± 1.135	**0.0366**

P-values ≤ 0.05 were considered statistically significant and are highlighted in bold.

### 2.5 Function of the DEGs validated in the strain collection

Six DEGs identified in the strain collection were related to the arginine pathways (3046, 3046b, 1815, 1255, 1256, and 1257, corresponding to the genes *argI*, *argF*, tRNA-Arg, *argC*, *argB*, and *argH*, respectively) (**Supplementary Table 2**). All of them were over-expressed in AIEC strains in the SN fraction compared to non-AIEC strains (p-value ≤ 0.042). Since differential expression in several genes related to the arginine pathway was statistically significant, three genes related to this amino acid (*adiA*, *adiC*, and *potE*) were studied in order to determine if this differential expression could be related with acid resistance. The genes *adiA* and *adiC* belong to the arginine-dependent acid resistance system, responsible of the arginine decarboxylation to agmatine and the pH-dependent arginine:agmatine transport. The gene *potE* codifies for the ornithine:putrescine antiporter that belongs to the ornithine-dependent acid resistance system (Charlier and Bervoets, 2019). Similar results were obtained for these genes, with all of them over-expressed in AIEC strains compared to non-AIEC in the SN condition, but in this case, the differences were not statistically significant (**Supplementary Table 4**).

Moreover, the genes 0036 (*tdcA*) and 3172b (*yjjB*) were over-expressed in AIEC strains in SN compared to non-AIEC strains. The *tdcA* gene encodes for a transcriptional regulator that activates the *tdc* operon and is related to serine degradation, while *yjjB* is a putative serine/threonine exporter.

Furthermore, five genes presented functions related to colanic acid biosynthesis and export (0413, 0414, 0418, 0420, and 0415 corresponding to *wza*, *wzb*, *wcaE*, *gmd*, and *wzc*, respectively). In non-AIEC strains, *wzc*, *wcaE*, and *gmd* were over-expressed at the A/I fraction compared to the SN, while *wzb* was over-expressed in AIEC SN compared to AIEC A/I and non-AIEC SN. However, no significant differences were observed, and more strains could be included to verify these findings.

Three genes were related to type 1 fimbriae (1395, 1396, 1397 corresponding to *fimC* precursor, *fimD*, and *fimF* precursor, respectively), all under-expressed in the SN fraction compared to A/I in non-AIEC strains. Furthermore, *fimH* was included in this analysis, and an increased expression in AIEC SN was observed compared to non-AIEC SN, with a mean of log_2_ RTA of 0.49 and -2.13, respectively (p-value = 0.019).

Moreover, three genes were associated with sorbitol biosynthesis (1689, 1690, and 1688 corresponding to *srlB*, *srlAE*, and *srlD*, respectively). They all presented statistically significant differences (p-value ≤ 0.0289 between SN and A/I fractions in non-AIEC strains.

Finally, *chiA* was also detected as a DEG. This gene encodes for a chitinase protein in the bacterial membrane and promotes the adhesion of the AIEC strains in IECs (Low et al., 2013). A significant increase in expression was found in non-AIEC A/I compared to non-AIEC SN (p-value = 0.017). Additionally, an over-expression of *chiA* was observed in AIEC in both SN and A/I conditions compared to non-AIEC strains, although no statistically significant differences were detected.

### 2.6 Assessment of DEGs as biomarkers for AIEC identification

**Figure 6:**
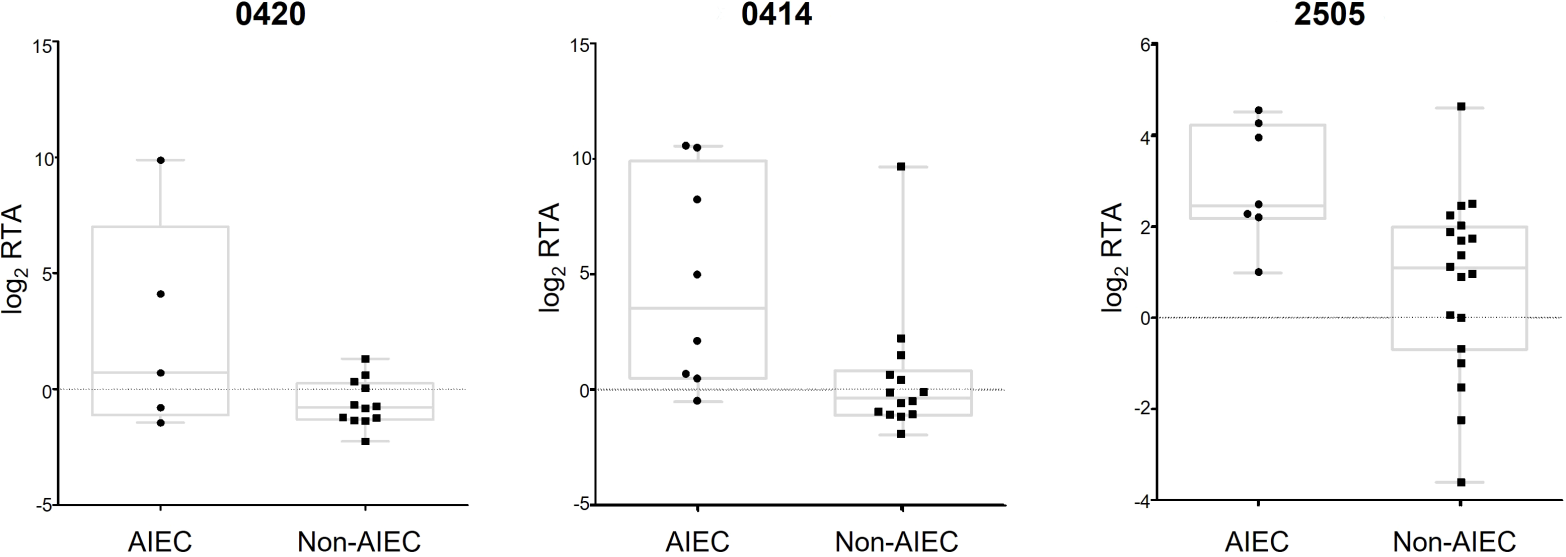
Boxplot of the genes that could predict AIEC phenotype with an accuracy >85% by binary logistic regression. Two (0414 and 2505) were selected as putative biomarkers, and 0420 was dismissed due to high dispersion. Log2 RTA values are represented for all the samples used in this study, classified by phenotype.

The applicability of DEGs as molecular markers for AIEC identification was assessed using binary logistic regression for each gene. The data analysis of SN samples revealed a list of 19 genes, each capable of distinguishing between AIEC and non-AIEC strains with an accuracy ≥ 77.1% (**Table 4**; **Supplementary Table 5**). Notably, in three cases, the quantification of a single gene reached an accuracy of 85.7% (**Figure 6**). Two of these genes are related to colanic acid biosynthesis (0414 and 0420), and the other (2505) is a transcriptional regulator cooper-dependent. However, the 0420 gene was dismissed as a candidate since a high statistical dispersion was observed (**Figure 6**), and no statistically significant differences in gene expression were observed between AIEC and non-AIEC strains when the t-test was performed in the strain collection. In the analysis executed with log_2_ RTA obtained in A/I samples, no molecular markers were found to distinguish between the two phenotypes.

**Table 4:** The binary logistic regression model for the putative molecular markers associated with the AIEC phenotype was calculated from the log_2_ RTA values for each DEG.

ID	Gene	Specificity (%)	Sensitivity (%)	Accuracy (%)	p-value	TP	TN	FP	FN
*chiA*	*chiA*	91.3	58.3	80.0	0.014	7	21	2	5
0036	*tdcA*	87.0	66.7	80.0	0.003	8	20	3	4
0413	*wza*	91.3	66.7	82.9	0.011	8	21	2	4
0414	*wzb*	95.7	66.7	85.7	0.006	8	22	1	4
0415	*wzc*	91.3	66.7	82.9	0.004	8	21	2	4
0420	*gmd*	91.3	75.0	85.7	0.014	9	21	2	3
1071	*nirB*	87.0	58.3	77.1	0.006	7	20	3	5
1255	*argC*	82.6	66.7	77.1	0.007	8	19	4	4
1256	*argB*	87.0	66.7	80.0	0.003	8	20	3	4
1257	*argH*	87.0	58.3	77.1	0.006	7	20	3	5
1395	*fimD*	95.7	58.3	82.9	0.016	7	22	1	5
1815	*UM146_RS05255*	91.3	66.7	82.9	0.003	8	21	2	4
1983	*ydjM*	91.3	66.7	82.9	0.002	8	21	2	4
2505	*cueR*	91.3	75.0	85.7	0.006	9	21	2	3
2523	*ffs*	82.6	75.0	80.0	0.010	9	19	4	3
2857	*pstA*	87.0	66.7	80.0	0.002	8	20	3	4
3029	*UM146_RS22055*	78.3	75.0	77.1	0.004	9	18	5	3
3163	*btsT*	87.0	75.0	82.9	0.009	9	20	3	3
3227	*UM146_RS24370*	87.0	58.3	77.1	0.015	7	20	3	5

TP, true positive; TN, true negative; FP, false positive; FN, false negative.

Gene expression values were those obtained only in the SN fraction. The percentage and values of successfully classified strains are indicated. Only genes with an accuracy >75% are shown.

## 3 Discussion

The AIEC pathotype is considered important in the inflamed gut of patients with CD, but its pathogenic mechanisms are not fully understood, and there are currently no molecular markers for its identification. This study aimed to identify genes involved in AIEC adhesion, invasion, and intracellular replication that could be used as markers for the molecular detection of the pathotype. Comparative transcriptomics was performed between two AIEC/non-AIEC strain pairs with identical pulsed-field gel electrophoresis (PFGE) patterns, reducing the probability of detecting DEGs inherent to the phylogenetic origin of the strains and increasing the likelihood of detecting genes specifically related to the AIEC phenotype. Gene expression was analyzed during IEC infection to ensure that genes involved in adhesion, invasion, and intracellular replication were expressed. RT-qPCR was used to quantify the expression level of DEGs in a larger collection of AIEC and non-AIEC strains from different phylogroups to validate the results. This approach identified genes associated with the AIEC phenotype and putative molecular markers that could be used for AIEC screening.

### 3.1 Differential gene expression in AIEC during intestinal epithelial cells infection

In the strain collection, 29 genes were differentially expressed between AIEC and non-AIEC strains. Surprisingly, most of these genes (28 out of 29) were found in the SN fraction before adhesion or invasion occurred. This unexpected result may be because the proportion of non-AIEC bacteria adhered to the IECs may have a similar gene expression pattern to AIEC adhered to IECs, and adhered bacteria are much more abundant than intracellular bacteria in these samples. However, the 42 genes differentially expressed between the SN and A/I fractions in the strain collection may be a source of new virulence factors involved in cell adhesion and invasion. Interestingly, 40 of these 42 genes were detected in non-AIEC strains, while only 11 were detected as differentially expressed in AIEC strains. This unexpected result may be explained by the fact that these virulence factors are constitutive genes or present some basal expression in AIEC strains, while in non-AIEC strains, they are only expressed after entering into contact with IECs.

The study of genes related to L-arginine biosynthesis caught our attention, particularly the genes *argI*, *argF*, *argC*, *argB*, and *argH*, which are regulated by ArgR, a repressor that acts in the presence of arginine. In the strain collection, these genes were over-expressed in AIEC in the SN fraction. When analyzed individually, these genes were over-expressed in 77.8%, 54.5%, 90.0%, 90.0%, and 100.0% of the AIEC strains, respectively. Interestingly, in a study by Elhenawy et al., *in vitro* and *in vivo* gene expression of the AIEC strain NRG857c was compared by comparative transcriptomics, and it was shown that genes involved in the arginine biosynthesis pathway are upregulated in the intestinal environment of animal models. Additionally, these genes are involved in AIEC survival *in vivo* and are essential for robust gut colonization, as mutants of *argBCGH* genes were significantly depleted from the murine intestine (Elhenawy et al., 2021).

On the other hand, statistically significant differences were observed in the gene *tdcA*, which was over-expressed in AIEC SN compared with non-AIEC SN and AIEC A/I. This gene is a transcriptional activator of the *tdc* operon, responsible for L-serine degradation. A previous study showed that several conditions associated with the inflamed gut, such as glucose starvation and oxygen and nitric oxide, lead to an up-regulation of these genes (Kitamoto et al., 2019).

In addition, AIEC strains have been observed to under-express genes involved in the biosynthesis of colanic acid building blocks (*wza*, *wzb*, *wcaE*, *gmd*, and *wzc*) in the A/I fraction compared to non-AIEC strains. Colanic acid is involved in developing mature biofilms but not in bacterial adhesion to different biotic and abiotic surfaces (Zhang and Poh, 2018; Navasa et al., 2019). The capsular polysaccharide colanic acid inhibits binding with several inert surfaces (Hanna et al., 2003). Therefore, the down-regulation of these genes observed in AIEC strains during IEC interaction may lead to increased bacterial adherence. Moreover, we observed that AIEC strains over-express these genes in the SN fraction compared to non-AIEC strains, although only *wza* and *wzb* showed statistically significant differences. Consistent with this, it has been demonstrated that colanic acid protects bacteria against complement-mediated killing and enhances *E. coli* motility (Lippolis et al., 2018).

The genes belonging to the *fim* operon (*fimC* precursor, *fimD* precursor, *fimF*, and *fimH*) were over-expressed in AIEC compared to non-AIEC strains in both fractions, although statistically significant differences were only observed in the SN fraction. When comparing the SN and A/I fractions, similar gene expressions were observed in AIEC strains, while in non-AIEC strains, an over-expression in the A/I fraction was seen. It is known that the expression of the *fim* operon is a phase variable and depends on the orientation of an invertible element that contains the promoter. Consistent with our findings, it has been observed that non-AIEC strains mainly present the OFF phase, avoiding the expression of the *fim* operon (Dreux et al., 2013). The *fim* operon encodes for the type 1 fimbriae, a well-known virulence factor for AIEC that interacts with the antigen-related cell adhesion molecule 6 (CEACAM6) located on the apical surface of epithelial cells. CEACAM6 expression is upregulated in CD patients, leading to increased interaction (Barnich and Darfeuille-Michaud, 2010). Furthermore, the over-expression of *fimH* observed in AIEC strains in this study may contribute to these interactions.

Another gene related to adhesion that was over-expressed in AIEC, especially in the SN fraction, was *chiA*. Furthermore, when comparing the SN and A/I fractions, AIEC strains presented similar expressions, while non-AIEC strains had higher expressions in the A/I fraction. This gene codes for a chitinase, an enzyme that hydrolyzes a long-chain polymer of N-acetylglucosamine called chitin. This protein interacts with human chitinases, which are, in turn, over-expressed in IECs during intestinal inflammation, promoting the adhesion of bacteria (Mizoguchi, 2006; Chen et al., 2011). Low et al. detected a specific interaction between a chitin-binding domain of bacterial ChiA and the N-glycosylation of asparagine 68 residue in mouse Chitinase 3-like-1 (Low et al., 2013). Furthermore, five polymorphisms were observed in a chitin-binding domain of LF82 that were not present in K-12 and were required for direct interaction with IECs. This variant observed in LF82 was found in 35.5% of AIEC strains and 7.4% of non-AIEC strains in the same strain collection (Camprubí-Font et al., 2019). Moreover, we observed an over-expression of the *chiA* gene in AIEC strains compared to non-AIEC strains, which may lead to increased interaction with human chitinases and enhance bacterial adhesion.

### 3.2 Biomarkers for AIEC molecular detection

Several genetic elements have been proposed as putative molecular markers for AIEC (Dogan et al., 2014; Deshpande et al., 2015; Desilets et al., 2016; Vazeille et al., 2016; Céspedes et al., 2017; Camprubí-Font et al., 2018). Desilets et al. suggested a biomarker based on the amplification of three genomic regions with an accuracy of 85%, although it was only present in B2 AIEC strains (Desilets et al., 2016). Vazeille et al. revealed an amplification of two genes that could predict the phenotype with an accuracy of 83% but low sensitivity (31%) (Vazeille et al., 2016). Recently, a molecular marker based on *chuA*, *eefC*, and *fitA* genes has been proposed, with a prevalence of 55.6–77.8% in AIEC strains, in contrast to other *E. coli* strains (14.3–17.5%) (Saitz et al., 2022). However, this was not AIEC-specific, and further analyses with geographically diverse *E. coli* strains are needed. Camprubí-Font et al. presented an algorithm based on SNPs sequencing with an accuracy of 81%, but it was not universal for all AIEC strains (Camprubí-Font et al., 2018, 2020). In our study, binary logistical regression analysis revealed two genes whose quantification presents higher sensitivities than other molecular markers previously suggested. In particular, this method can predict the AIEC phenotype with an accuracy of 85.7% in our strain collection. This would represent a faster, more reproducible, and putatively automatable method for AIEC identification. Nonetheless, further studies, including geographically diverse AIEC strains, are needed to determine if these biomarkers are universal. Other *E. coli* pathotypes, such as ExPEC, must be included to ensure these are AIEC-specific. Apart from testing the specificity *in vitro* using external strain collections a further step will be to test the applicability of the biomarkers directly in clinical samples. Gene expression will certainly change in *in vivo* conditions; however, we hypothesize that if the genes are relevant for AIEC pathogenicity it is probable these genes will be also overexpressed *in vivo*. However, further studies are needed to evaluate the applicability and usefulness of these biomarkers in disease management.

### 3.3 Concluding remark

Our study has increased our knowledge of the molecular mechanisms involved in AIEC pathogenicity. Gene expression levels of AIEC and non-AIEC strains in both SN and A/I fractions have revealed genes and pathways that may be involved in the adhesion and invasion of AIEC to IECs. These results provide a foundation for future investigations of therapeutic targets against AIEC colonization. Furthermore, we have proposed two genes as biomarkers to identify AIEC strains based on quantifying their expression levels. Subsequent studies to validate the distribution of these biomarkers in a geographically diverse collection of AIEC strains and their usefulness in clinical samples are of great interest.

## 4 Methods

### 4.1 Strains of study, cell line and growth conditions

Two previously isolated and characterized AIEC/non-AIEC strain pairs were used in this study. These pairs correspond to strains with similar genome structure, gene content, identical PFGE patterns, and sequence types, but with a different AIEC phenotype (Martinez-Medina et al., 2009; Camprubí-Font et al., 2018). The AIEC07/ECG04 strain pair was isolated from the ileum of a control patient and belonged to the B1 phylogroup, while the AIEC17/ECG28 strain pair was obtained from the colon of a CD patient and corresponded to the D phylogroup.

The Intestine-407 epithelial cell line (I-407; ATCC CCL-6) was maintained in an atmosphere containing 5% CO_2_ at 37°C with EMEM medium (Lonza, Switzerland) supplemented with 10% fetal bovine serum (FBS; Gibco, USA), 1% antibiotic-antimycotic (Gibco, USA), 1% glutamine (Gibco, USA), 1% minimum essential medium non-essential amino acids (Gibco, USA), and 1% minimum essential medium vitamins (Gibco, USA).

### 4.2 Infection of intestinal epithelial cells

T75 flasks were seeded with 2 × 10^7^ I-407 cells and incubated for 20 h. Cells were washed twice with 10 mL of phosphate-buffered saline (PBS; Lonza, Switzerland), and a cell culture medium composed of EMEM (Lonza, Switzerland) with 10% heat-deactivated FBS (Gibco, USA) was added. Infection of cells was performed at a multiplicity of infection (MOI) of 100, with bacteria growing exponentially in LB broth (OD600 = 0.625). After 4 h of incubation at 37°C with 5% CO_2_, the supernatant (SN fraction) was collected, and the eukaryotic cells containing adhered and invading bacteria (A/I fraction) were washed twice with the cell culture medium and recovered with a scraper. Both fractions were immediately kept on ice and centrifuged at 4°C for 10 min at 3000 × g. The supernatant was discarded, and the pellet was washed with 0.5 mL of PBS.

### 4.3 mRNA extraction and purification

Both the SN and A/I fractions were processed equally. The TRIzol Max Bacterial Isolation kit with Max Bacterial Enhancement reagent (Invitrogen, USA) was used to obtain total RNA, following the manufacturer’s instructions with the following modifications: an incubation of 15 min at room temperature was done after chloroform addition, and overnight precipitation at -20°C was performed after the addition of isopropanol. Subsequently, a DNase I treatment (Thermo Scientific, USA) was applied using 1 U of DNase for 5 µg of RNA. The samples obtained at this point were named ‘non-purified’ samples.

To begin RNA purification, mRNA containing a polyA tail, 18S and 28S eukaryote rRNAs were removed with the MICROBEnrich kit (Thermo Scientific, USA) using Oligo MagBeads. For all samples, at least 25 µg of total RNA in a maximum of 30 µl were treated with this kit. If the RNA concentration was insufficient, an additional precipitation step was carried out as recommended in the kit instructions. The Ribo-Zero Magnetic Gold kit (epidemiology) (Illumina, USA) was used on 2.5 µg of sample RNA to remove all rRNAs and a part of miRNA and tRNA, obtaining mainly purified prokaryotic mRNA. The samples obtained at this point were named ‘purified’ samples.

RNA quantification was done before and after each kit with a Nanodrop spectrophotometer and a Qubit Fluorometer with RNA HS Assay kit (Thermo Scientific, USA). Additionally, RNA quality was verified with a denaturing agarose gel and/or the Total Prokaryotic RNA program of the Agilent RNA 6000 Nano Chip Bioanalyzer (Agilent, USA).

### 4.4 RNA-seq and comparative transcriptomics

The TruSeq Stranded mRNA method (Illumina, USA) was used for cDNA synthesis. Two SN samples and one A/I sample were sequenced for each strain by Illumina Miseq. The sequencing depth varied according to the fraction: 10 million reads for samples mainly composed of bacteria (SN fraction) and 200 million reads for samples from infected cells (A/I fraction).

Sequence reads were analyzed using FastQC,(Andrews, 2010) trimmed, and mapped to the UM146 AIEC reference genome (NC_017632) with the TopHat (Trapnell et al., 2009). Transcripts were assembled using Cufflinks (Trapnell et al., 2012), merged and compared to UM146 by Cuffmerge (Trapnell et al., 2012). Finally, normalization and differential expression analysis were done using Cuffdiff (Trapnell et al., 2012). Gene expression levels were presented as fragments per kilobase of transcript per million mapped reads (FPKM). Four comparisons were performed, in which AIEC transcripts were compared with non-AIEC transcripts for each condition and each pair. Those genes with p ≤ 0.05 were considered differentially expressed genes (DEGs). Fold change was calculated for each DEG by dividing the FPKM value of non-AIEC by the FPKM value of AIEC. All the DEGs were classified into eight functional categories by BlastX and extensive bibliographic research.

### 4.5 Bacterial strains and RNA extraction for validation of differentially expressed genes

All DEGs identified in the comparative transcriptomics analysis were studied in a phylogenetically diverse *E. coli* strain collection to determine whether these expression differences were widely distributed among AIEC strains and could be used as molecular biomarkers for AIEC identification. The strain collection included 13 AIEC and 22 non-AIEC strains isolated from ileum and colon biopsies of patients with clinically confirmed inflammatory bowel disease and from control subjects (Martinez-Medina et al., 2009). The commensal strain K-12 C600 and the reference AIEC strain LF82 were added to the collection of tested strains. Overall, the strain collection was composed of strains from phylogroups A (16.2%), B1 (8.1%), B2 (59.4%), D (13.5%), and an atypical strain (2.7%) (**Supplementary Table 6**). The strains were phenotypically characterized, and all genomes had been previously sequenced (Martinez-Medina et al., 2020).

The infection and RNA extraction protocols were the same as those described for the strain pairs in the previous sections (Infection of intestinal epithelial cells and mRNA extraction and purification). However, the samples in this part of the analysis were not treated with the MICROBEnrich or Ribo-Zero Magnetic Gold kit, so non-purified RNA extracts were obtained from the SN and A/I fractions. The strain pairs used for RNA-seq were also included in this part of the analysis, so gene expression levels could be compared between purified and non-purified samples for the AIEC17, AIEC07, ECG28, and ECG04 strains.

### 4.6 RT-qPCR for gene expression quantification

The previously identified DEGs were quantified in the same sequenced samples to validate RNA-seq results, as well as in non-purified strain pair extracts and a collection of AIEC and non-AIEC strains.

First, 1.5 µg of RNA was reverse transcribed using the High-Capacity cDNA Reverse Transcription Kit (Applied Biosystems, USA) and the RNase Inhibitor (Life Technologies, USA) in a final volume of 20 µL. Since the SN samples of the strain pairs were studied in duplicate, they were mixed equally before cDNA synthesis.

Gene-specific primers were designed with the support of Primer3 0.4.0 (**Supplementary Table 7**). The considered parameters were primer size (15–30 nucleotides), GC content (30–80%), amplicon size (<200 base pairs), and primer melting temperature (55–60°C). Secondary structures were estimated with NetPrimer (PREMIER Biosoft International, Palo Alto, CA). The specificity of the primers in Bacteria was assessed by performing *in silico* PCR amplifications at http://insilico.ehu.es/PCR/. No amplification of the human genome was verified through two databases (UCSC In-Silico PCR and BIOTECH In Silico PCR).

RT-qPCR was performed in a 96 × 96 microfluidic array IFC chip on a BioMark™ system (Fluidigm, USA). The Fluidigm loading kit-10 IFCs (BMK-M10-96.96) was used following the manufacturer’s instructions. Before amplification, a cycle of 10 min at 95°C followed by 16 cycles of 15 s at 95°C and 4 min at 60°C was performed. Three technical replicates were analyzed for each sample.

The A/I fraction of the LF82 AIEC reference strain was included in the analysis of each gene with a five-fold dilution series (1/4, 1/20, 1/100, 1/500, 1/2500) to determine the calibration curve and amplification efficiency (*E* = 10 ^(-1/slope)^) (Svec et al., 2015). To normalize data and compare samples with different concentrations, *gapA* was selected as the housekeeping gene (Jandu et al., 2009).

The relative transcript abundance (RTA) of mRNA for each DEG was calculated with the equation (Pfaffl, 2012):


$$\mathrm{RTA}=\frac{E^{▵\mathrm{Ct}\;\left(\mathrm{reference}-\mathrm{sample}\right)}\left(\mathrm{target}\;\mathrm{gene}\right)}{E^{▵Ct\;\left(\mathrm{reference}-\mathrm{sample}\right)}\left(\mathrm{housekeeping}\;\mathrm{gene}\;\mathrm{gapA}\right)}$$

The respective AIEC strain was applied as a reference in the study of the strain pairs. Since RTA was calculated for the non-AIEC strain using the respective AIEC as a reference in the strain pairs, negative log_2_ RTA (values<1) indicate over-expression in AIEC, while positive log_2_ RTA (>1 value) indicates under-expression in AIEC. In the strain collection, LF82 A/I sample was used as reference to calculate RTA values. Therefore, negative log_2_ RTA a lower expression of the sample compared with LF82 A/I while positive log_2_ RTA indicates a higher expression.

### 4.7 Statistical analysis of RT-qPCR data

The level of correlation between the log_2_ fold change values obtained from the comparative transcriptomics of RNA-seq data and the log_2_ RTA values obtained from Fluidigm analysis was determined using Spearman’s correlation. The effect of mRNA purification on gene expression ratio in RNA extracts was studied by comparing log_2_ RTA values of purified and non-purified samples of the strain pairs using a paired t-test.

To identify which DEGs were differentially expressed between AIEC and non-AIEC phenotypes, gene expression levels were compared between AIEC and non-AIEC strains of the whole strain collection using a Mann-Whitney U test for non-parametric data or a t-test for parametric data. In this analysis, the mean of log_2_ RTA values was compared between the AIEC and the non-AIEC groups for each DEG. Previously, a Shapiro-Wilk test was performed to assess if the variables were normally distributed, and the Levene test was used to assess homoscedasticity. Gene expression levels were also compared between SN and A/I fractions using a paired t-test for each phenotype. A chi-squared test was used for each gene to compare the detection frequency between AIEC and non-AIEC strains.

To determine which DEGs could be used to predict the AIEC phenotype, a binary logistic regression analysis was performed for each DEG individually using the log_2_ RTA values obtained. Before the analysis, all missing values were replaced. Samples with no amplification of the DEG and a Ct*_gapA_* < 18 were considered to have a correct RNA concentration but an undetectable gene expression. To give a value for these samples, the log_2_ RTA was calculated by replacing the no-amplified Ct with 25, which is the detection limit of Fluidigm. Samples with a Ct*_gapA_* > 18 were considered not concentrated enough to ensure accurate quantification of genes with low expression levels. In this case, the missing values were substituted with the mean value of log_2_ RTA for each DEG according to pathotype to avoid bias. Only genes amplified in >60% of strains were analyzed, and those with high predictive potential for the AIEC phenotype were selected based on their sensitivity, specificity, and accuracy.

All statistical analyses were performed with either SPSS (IBM Corp. Released 2010. IBM SPSS Statistics for Windows, Version 19.0. Armonk, NY: IBM Corp.) or GraphPad (GraphPad Prism version 5.00 for Windows, GraphPad Software, La Jolla California USA). For all analyses, a p-value ≤ 0.05 was considered statistically significant.

## Data availability statement

The raw data supporting the conclusions of this article are available from the Catalan Open Research Area (CORA) repository, with the following DOIs: https://doi.org/10.34810/data699 and https://doi.org/10.34810/data698.

## Author contributions

MM-M designed the study. QB-R, CC-F, and ML-S obtained the data. QB-R performed the statistical analysis. QB-R, CC-F, ML-S, and MM-M performed data interpretation. QB-R drafted the manuscript. CC-F, ML-S, and MM-M revised the manuscript. MM-M obtained funding. All authors contributed to the article and approved the submitted version.
